# Inhibition of *Plasmodium falciparum* Field Isolates-Mediated Endothelial Cell Apoptosis by Fasudil: Therapeutic Implications for Severe Malaria

**DOI:** 10.1371/journal.pone.0013221

**Published:** 2010-10-07

**Authors:** Estelle S. Zang-Edou, Ulrick Bisvigou, Zacharie Taoufiq, Faustin Lékoulou, Jean Bernard Lékana-Douki, Yves Traoré, Dominique Mazier, Fousseyni S. Touré-Ndouo

**Affiliations:** 1 Centre International de Recherches Médicales de Franceville (CIRMF), Franceville, Gabon; 2 Institut National de la Santé et de la Recherche Médicale (INSERM) U511, Paris, France; 3 Université de Ouagadougou, Ouagadougou, Burkina Faso; 4 Université Pierre et Marie Curie Paris 6, UMR S511, Paris, France; 5 Assistance Publique-Hôpitaux de Paris (AP-HP), Groupe hospitalier Pitié-Salpêtrière, Service Parasitologie-Mycologie, Paris, France; Walter and Eliza Hall Institute of Medical Research, Australia

## Abstract

*Plasmodium falciparum* infection can abruptly progress to severe malaria, a life-threatening complication resulting from sequestration of parasitized red blood cells (PRBC) in the microvasculature of various organs such as the brain and lungs. PRBC adhesion can induce endothelial cell (EC) activation and apoptosis, thereby disrupting the blood-brain barrier. Moreover, hemozoin, the malarial pigment, induces the erythroid precursor apoptosis. Despite the current efficiency of antimalarial drugs in killing parasites, severe malaria still causes up to one million deaths every year. A new strategy targeting both parasite elimination and EC protection is urgently needed in the field. Recently, a rho-kinase inhibitior Fasudil, a drug already in clinical use in humans for cardio- and neuro-vascular diseases, was successfully tested on laboratory strains of *P. falciparum* to protect and to reverse damages of the endothelium. We therefore assessed herein whether Fasudil would have a similar efficiency on *P. falciparum* taken directly from malaria patients using contact and non-contact experiments. Seven (23.3%) of 30 PRBC preparations from different patients were apoptogenic, four (13.3%) acting by cytoadherence and three (10%) via soluble factors. None of the apoptogenic PRBC preparations used both mechanisms indicating a possible mutual exclusion of signal transduction ligand. Three PRBC preparations (42.9%) induced EC apoptosis by cytoadherence after 4 h of coculture (“rapid transducers”), and four (57.1%) after a minimum of 24 h (“slow transducers”). The intensity of apoptosis increased with time. Interestingly, Fasudil inhibited EC apoptosis mediated both by cell-cell contact and by soluble factors but did not affect PRBC cytoadherence. Fasudil was found to be able to prevent endothelium apoptosis from all the *P. falciparum* isolates tested. Our data provide evidence of the strong anti-apoptogenic effect of Fasudil and show that endothelial cell-*P. falciparum* interactions are more complicated than previously thought. These findings may warrant clinical trials of Fasudil in severe malaria management.

## Introduction

*Plasmodium falciparum* malaria remains a major life-threatening parasitic disease, killing about a million people each year, mainly in sub-Saharan Africa [1], [2], [3], [4]. Young children and pregnant women are particularly vulnerable. Why some non immune individuals die whereas others have uncomplicated or even asymptomatic infection is not known [5].

*P. falciparum* initially infects the liver before undergoing intrerythrocytic development through an asexual replication cycle [6]. This infection can progress unpredictably to severe forms, notably including anemia and cerebral malaria (CM) [7].

CM accounts for a significant proportion of the morbidity and mortality associated with malaria in children less than five years. Despite a massive amount of experimental and clinical work, the pathophysiologic mechanisms of this complication are poorly understood, although several studies implicate sequestration of *P. falciparum*-parasitized red blood cells (PRBC) within the brain [8], [9], [10], [11], [12]. This sequestration is characterized by PRBC adhesion (or cytoadherence), agglutination and rosetting [13], [10], [14]. Cytoadherence of PRBC to host endothelial cells (EC) in brain and lung capillaries can obstruct the microvasculature, a phenomenon accompanied by changes in the T cell repertoire and by cytokine production [15]. This cytoadherence is mediated by EC receptors such as CD36, intracellular adhesion molecule 1 (ICAM1), vascular cellular adhesion molecule 1 (VCAM1), CD31, integrins, thrombospondin, E-selectin and chondroitin sufate A [16], [17], PRBC adhesion can induce over-expression of inflammatory cytokines [15], [18], coagulation factors [19] and EC apoptosis [20], [21]. Blood-brain barrier (BBB) impairment associated with a direct cytotoxic effect of PRBC on EC has also been observed [22], [23]. It has been postulated that PRBC-mediated EC apoptosis could amplify BBB dysfunction [20]. More recently it has been demonstrated that hemozoin, the malarial pigment induces the erythroid precursor apoptosis [24].

Human lung endothelial cells (HLEC) are widely used to study the pathophysiology of *P. falciparum* malaria in vitro [25]. Using HLEC/PRBC coculture, we have previously shown that EC apoptosis triggered by cytoadherence may lead to neurological complications in *P. falciparum* malaria; however, only some *P. falciparum* isolates induced EC apoptosis [26]. We have also identified new PRBC ligands (Plasmodium apoptosis–linked pathogenicity factors, PALPF) associated with parasite apoptogenicity [27]. Although most of these PALPF are transmembrane proteins, only some are involved in parasite cytoadherence.

Parasite elimination is more beneficial in controlling the pathology. Host response to the infection is critical for CM development. However, the presence of the parasite is not harmless during a CM case, when in fact, it likely triggers the host response that promotes disease. Conventional emergency treatment is based on intravenous administration of antimalarial drugs such as quinine and artemisinin derivatives [28]. Despite effective parasite clearance, up to 20% of patients die and others have permanent sequelae [29], [30]. A new strategy combining parasite eradication and EC protection is therefore needed.

Rho kinase (ROCK), a serine threonine-specific protein kinase, is activated when it binds to GTP and is inactivated when it binds to GDP. Rho kinase phosphorylates many substrate proteins [31] and plays a key role in EC signal transduction through receptors such as ICAM1, VCAM1 and selectins [32]. Rho kinases are involved in PRBC–EC interactions: PRBC adhesion to EC activates the Rho kinase pathway, leading to EC apoptosis [33].

Fasudil (HA-1077) is a Rho kinase inhibitor that attenuates NF-kappa B activation [34]. Fasudil has been marketed since 1995 in Japan for the treatment of ischemic stroke [35]. Recently, it was shown that EC apoptosis can be inhibited by Fasudil [34], but these results were obtained only with in vitro-adapted parasite strains, although one of which was isolated from a CM patient. There are no relevant data on field isolates.

Here we examined the effect of fasudil on EC apoptosis mediated by RBC infected by field isolates. Two approaches were used: 1) contact coculture, to measure apoptosis mediated by cytoadherence; and 2) non contact experiments to examine the role of diffusible stimuli. We then examined the effect of fasudil on both mechanisms of EC apoptosis.

## Results

### Patients

Three hundred symptomatic children aged from 1 to 14 years were recruited for the study. Fifty-four children (18%) were found to be infected with *P. falciparum*. There were 53 cases of uncomplicated malaria and one case of severe malaria (hyperparasitemia: 26%) in the WHO classification.

### Parasites

Parasitemia ranged from 0.3% to 26% (table 1 and 2). Thirty of the 54 isolates were selected for further study. After enrichment using Plasmagel or gelatin flotation, PRBC specimens were found to be almost exclusively at the schizont stage and parasitemia was between 4% and 55% (data not shown).

**Table 1:** *Plasmodium falciparum* parasitized red blood cells (PRBC)-mediated human lung endothelial cells (HLEC) apoptosis induction and inhibition by Fasudil: Seventeen PRBC from F747 to F625 were cocultured in contact conditions (cytoadherence).

Pf isolates	Para.(%)	Nb* Schiz. (x10^6^)	Cyto. Per 1000 HLEC	Apoptosis induction/inhibition (OD)			
				4 hours		24 hours	
				Fasudil−	Fasudil+	Fasudil−	Fasudil+
F747	1.3	8	29	0.36	0.29	1.7	1.2
**F463**	1	-	199	**3**	**2**		
F464	3	-	3	2.23	0.61	2.5	2
**F803**	3.2	-	29	**3.1**	**1.67**	**18**	**4.8**
F856	0.3	-	10	0.82	0.81	1.10	0.9
F862	1.5	-	12	1.21	1.17	2.15	1.
F949	3.4	-	3	1.01	0.85		
**F126**	2	-	79	**4.71**	**1.56**	**28.27**	**7.4**
F528	1.3	-	202	0.83	0.62	1.5	1.2
F969	4	-	200	1.28	0.72	1.49	0.91
F995	3.5	-	2	0.86	0.43	1.54	0.73
F2008	2	-	4	0.98	0.89	2	1.8
**F127** **	26	-		0.79	0.58	**4.12**	**1.78**
F593	3	-	200	0.27	0.17	0.7	0.68
F609	2	-	150	0.49	0.33	0.5	0.16
F619	5	-	50	0.35	0.24	2.1	0.4
F625	4	-	100	0.5	0.28	1.69	1.69

Para., parasitemia; Nb, number; schiz. Schizont;

*(8×10^6^); Cyto., cytoadherence; OD., optical density. In red colour OD values of apoptosis positive and inhibition by Fasudil.

**Hyperparasitemia (Severe malaria case).

**Table 2:** *Plasmodium falciparum* parasitized red blood cells (PRBC)-mediated human lung endothelial cells (HLEC) apoptosis induction and inhibition by Fasudil: Ten PRBC from F528 to F625 were cocultured in non contact experiments.

Pf isolates	Para. (%)	Nb* schiz. (x10^6^)	Apoptosis induction/inhibition (OD)			
			4 hours		24 hours	
			Fasudil−	Fasudil+	Fasudil−	Fasudil+
**F528**	1.3	200	0.52	0.61	**3.35**	**1.12**
F2008	2	-	0.82	0.4	1.16	0.92
F126	2		0.55	0.29	0.56	0.52
F127**	26	-	0.66	0.51	0.79	0.58
**F969**	4	-	0.93	0.70	**3.57**	**1.08**
F593	3	-	0.98	0.39	0.8	0.74
**F995**	3.5	-	0.74	0.65	**3.15**	**1.02**
F609	2	-	0.66	0.61	0.5	0.3
F619	5	-			1.03	1.02
F625	4	-			1.03	1.03

Pf, *Plasmodium falciparum*; Para., parasitemia; Nb, number; schiz. Schizont;

*(24×10^6^); Cyto., cytoadherence; OD, optical density. In red colour OD values of apoptosis positive and inhibition by Fasudil.

**Hyperparasitemia (Severe malaria case).

### Cytoadherence

The number of schizonts in each PRBC preparation used in the cytoadherence tests was estimated at 24×10^6^. Binding assays were carried out with 17 PRBC samples. The adhesion was determined for 16 PRBC with values ranging from 2 to 202 PRBC per 1000 HLEC (Table 1). The number of adherent uninfected red blood cells was negligible. Fasudil had no effect on the PRBC adherence (data not shown).

### Apoptosis

The number of schizonts in each PRBC sample used in apoptosis assays was approximately 8×10^6^ and 200×10^6^ per well respectively for contact and non contact experiments.

#### Contact coculture

**Figure 1:**
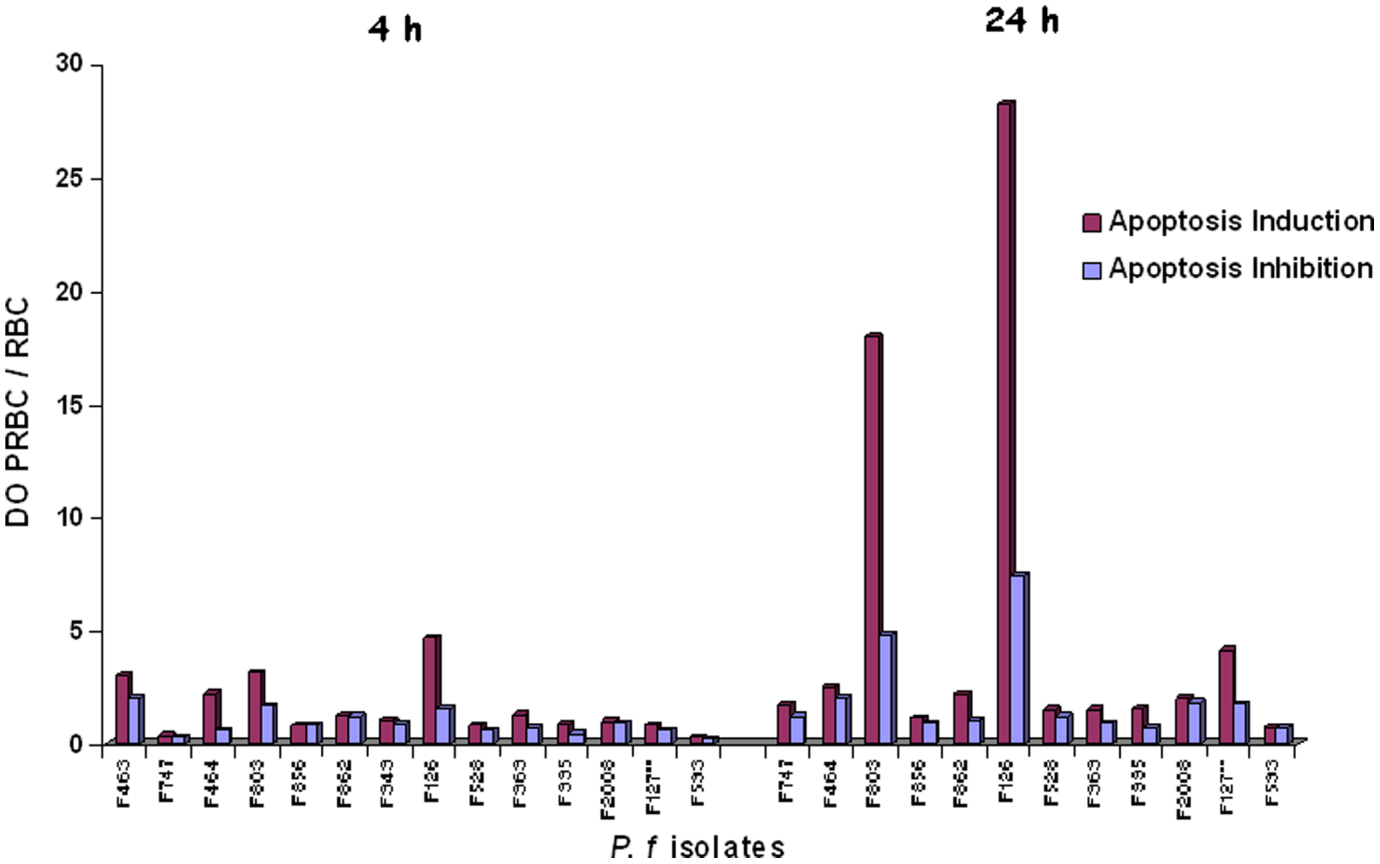
PRBC cytoadherence-mediated HLEC apoptosis and inhibition by Fasudil.

Thirty PRBC samples were each tested in quadruplicate for their capacity to induce HLEC apoptosis via cytoadherence. Four PRBC samples (13.1%) were found to trigger HLEC apoptosis in these conditions (Table 1, Figure 1). In three cases (samples F463, F803 and F126), EC apoptosis occurred within 4 h of coincubation, with OD ratios ranging from 3.0 to 4.7 (mean 3.6).The OD ratios for F803- and F126-activated HLEC were 18 and 28.27 respectively (mean 23.1) after 24 h of coculture (Table 1). With F127-activated HLEC, apoptosis was observed after at least 24 h of coincubation, with an OD ratio of 4.12. The mean OD ratio for F803-, F126- and F127-activated HLEC after 24 h of coculture was 16.8. These results show that apoptosis increases significantly with time (P<0.05).

#### Non contact coculture

**Figure 2:**
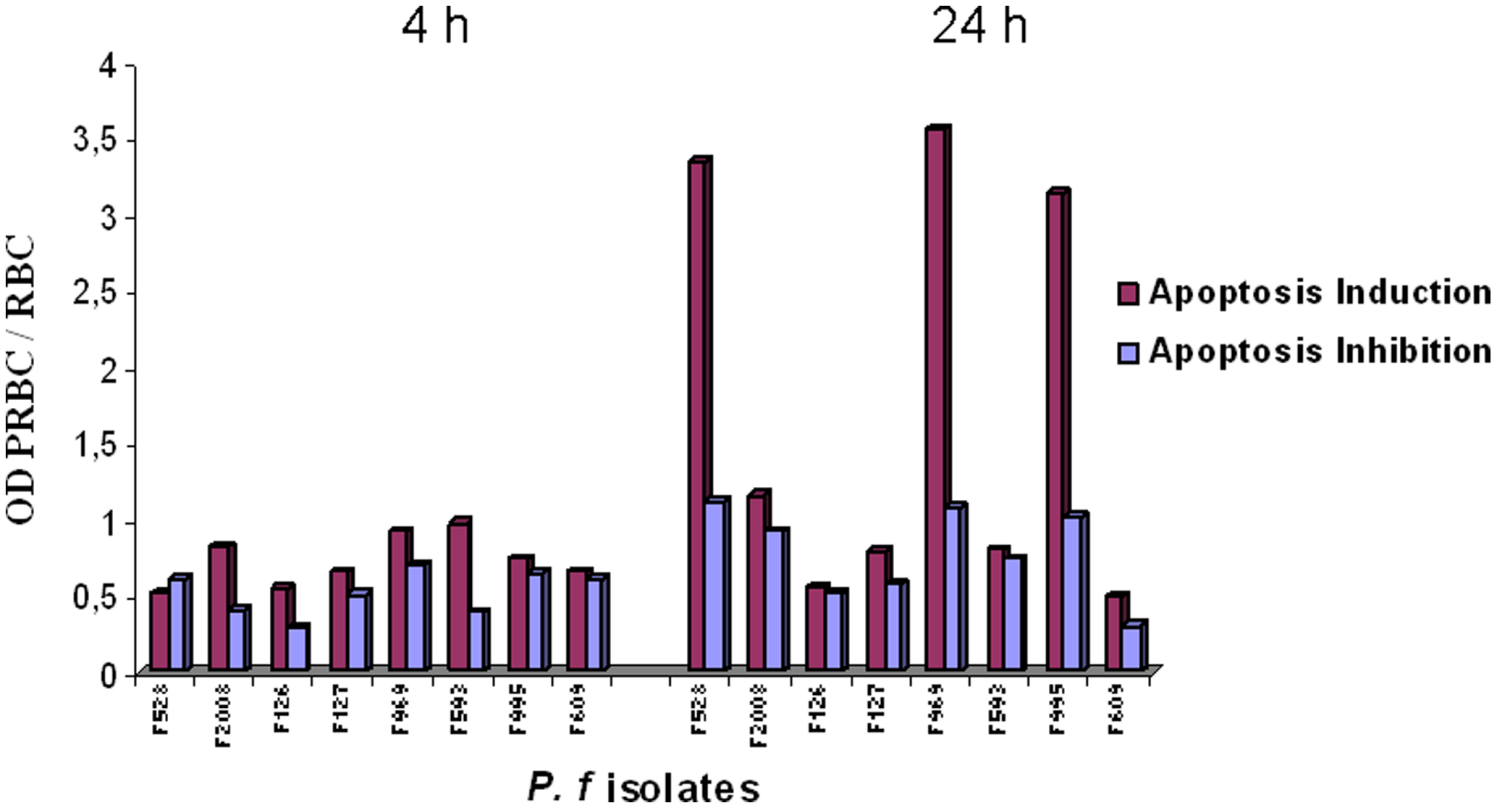
PRBC soluble factors-mediated HLEC apoptosis and inhibition by Fasudil.

To determine whether PRBC could induce HLEC apoptosis via the release of diffusible soluble stimuli, without cell-cell contact, the same 30 PRBC specimens were coincubated with HLEC in Transwells. No HLEC apoptosis was observed after 4 h of coculture, but three specimens (10%) (samples F528, F969 and F995) induced HLEC apoptosis after 24 h, with respective OD ratios of 3.35, 3.57 and 3.15 (mean 3.36) (Table 2, Figure 2).

### Effect of Fasudil on PRBC-mediated HLEC apoptosis

HLEC were concomitantly exposed to PRBC suspensions containing 30 µM Fasudil in contact (96-well plates) (Table 1, Figure 1) and non contact (Transwell) conditions (Table 2, Figure 2) for 4 h and 24 h.

After 4 h in contact conditions, Fasudil inhibited F463-, F803- and F126-activated HLEC apoptosis (OD), relative to untreated controls, from 3.0 to 2.0 (34%); 3.1 to 1.67 (46.2%); and 4.7 to 1.5 (67%) respectively (mean inhibition 49.06%). At 24 h, F803-, F126- and F127-induced HLEC apoptosis was significantly reduced by Fasudil, from 18 to 4.8 (73.3%); 28.27 to 7.4 (73.8%) and 4.1 to 1.78 (56.8%), respectively (mean inhibition 68%).

In non contact conditions, HLEC apoptosis induced by F528, F969 and F995 was reduced by Fasudil from 3.35 to 1.12 (66.5%); 3.57 to 1.08 (69.7%) and 3.1 to 1.0 (67.6%), respectively (mean inhibition 68%).

Overall, 7 (23.33%) apoptogenic PRBC preparations were identified, 4 (13.3%) requiring cytoadherence (Table 2, Figure 1) and 3 (10%) capable of acting via soluble factors (Table 2, Figure 2). Fasudil thus significantly reduced *P. falciparum*-mediated HLEC apoptosis, by an average of 68% after 24 h, in both contact and non contact conditions.

## Discussion

Knowledge of the pathophysiological mechanisms of severe malaria, and especially parasite ligands and host cell receptors, is crucial for designing specific pathway inhibitors. We have previously investigated signal transduction pathways involved in the pathogenicity of *P. falciparum* malaria by using a coculture model, and identified parasite factors linked to endothelial cell apoptosis termed *Plasmodium* apoptosis-linked pathogenicitty factors (PALPF) [27]. In 2008, Taouficq et al. demonstrated that Rho kinase inhibition by Fasudil reduced HLEC apoptosis induced by *P. falciparum*.

Here we examined the effect of fasudil on PRBC-mediated HLEC apoptosis, using *P. falciparum* isolates from symptomatic patients, in contact and non contact conditions. PRBC-mediated HLEC apoptosis has previously been studied by means of transmission electron microscopy, annexin V assay, caspase activity assay and nucleosome release ELISA [20].

We found that 23.33% of *P. falciparum* field isolates triggered HLEC apoptosis, 13.33% acting via cytoadherence and 10% via parasite-derived soluble factors. We believe that field isolates have different behaviour which remains intrinsic to the parasites and influenced by environment (vector, host, ecology). Twenty-nine isolates (97%) were from children with uncomplicated malaria (Table). We have previously observed a relation between HLEC apoptosis and severe malaria (especially neurological manifestations) and shown that HLEC apoptosis can be induced by PRBC from patients with uncomplicated malaria [26] and even from asymptomatic individuals (Touré personal data). These previous results suggested that uncomplicated or asymptomatic patients harboring the apoptogenic isolates may progress more rapidly to severe disease but the subsequent risk of developing such severe manifestations cannot be studied in children, since for ethical reasons treatment is started immediately after diagnosis.

Here we show, for the first time, that soluble factors released by *P. falciparum*-infected erythrocytes can trigger HLEC apoptosis, in line with the results obtained by Wilson in 2008 with human brain endothelial cells, but conflicting with our previous observations suggesting that direct contact between PRBC and HLEC was required for apoptosis [20], [26], [33]. This discrepancy may be explained by differences in the parasite strains and experimental approaches used. Indeed, in our previous work, we only used contact coculture, with PRBC isolated from symptomatic children contrasting with Pino and Taouficq who almost exclusively used PRBC reference strains 3D7 and F12 but in both contact and non contact conditions.

Our results show that HLEC apoptosis triggered by PRBC cytoadherence is detectable after only 4 h of coculture with most apoptogenic *P. falciparum* strains, and after 24 h for a further minority of strains. We have already demonstrated that the capacity of PRBC to trigger HLEC apoptosis is governed by genes encoding proteins essential for apoptosis induction [26], [27]. As the same number of PRBC for each isolate was used in our experiments, this difference in the timing of apoptosis onset is likely due to the nature and expression kinetics of the *Plasmodium* apoptosis-linked pathogenicity factors (PALPF) involved. We postulate that rapid transducer ligands (which induce EC apoptosis in 4 h) and slow transducer ligands (which induce EC apoptosis in 24 h) may exist. If so, the nature or affinities of HLEC receptors recognized by the two categories of ligands are also likely to be different. In “contact” experiment, after 24 h coculture both stimuli (cytoadherence and soluble factors) may be implicated in apoptosis induction. It is possible they act in synergy. Nevertheless, our data clearly indicate that none of the apoptogenic PRBC preparations used both mechanisms suggesting a possible mutually exclusion of signal transduction ligand. By contrast, in “non contact” only soluble factors released by parasite coculture medium can specifically induce EC apoptosis. We also found in contact experiments that the intensity of EC apoptosis increased with time since the mean OD from EC apoptosis within 24 h is significantly higher than the mean OD from EC apoptosis occurring in 4 h. Also, the apoptosis index of F803-activated HLEC rose from 3.1 at 4 h to 18 at 24 h, i.e. by factor 5.8. This index for F126-activated HLEC rose by a factor of 6. It is also interesting to notice that this intensity varies between various PRBC and that seems to be due to the level of expression of PALPF. By contrast, HLEC apoptosis induced by soluble factors was only detected after at least 24 h. Perhaps this delay is related to clinical threshold of the parasite-derived stimuli released in the coculture medium.

The EC signals triggered by PRBC adhesion are unclear. Nevertheless, PRBC adhesion modulates HLEC expression of tumor necrosis factor-α superfamily genes (Fas, Fas L, and DR-6) and apoptosis-related genes (Bad, Bax, caspases and iNOS etc.) [20], [21]. PRBC adhesion to EC directly activates the Rho kinase signaling pathway and induces the production of reactive oxygen species (ROS) by endothelial cells, both pathways potentially leading to cell death [20], [36]. Nitric oxyde (NO) overproduction can induce cytosolic release of mitochondrial cytochrome c, which also triggers caspase activation [37], [38]. Jimenez et al. found that binding of the CD36 ligand thrombospondin-1 was sufficient to induce EC apoptosis mediated by p59/fyn and caspases [39]. Finally it has been suggested that CD36 and ICAM-1 are the main EC receptors used by *P. falciparum in vivo* to promote parasite survival and escape the immune system [40], [41]. It is possible that PALPF use these receptors to induce EC apoptosis. All these works testified that the signal transduction properties from adhered PRBC are being understood, but that triggered by PRBC-derived soluble factors remain to be elucidated. However, it is possible that the two stimuli have the same signaling properties. Indeed, soluble factors or macromolecules released into the coculture medium may be able to recognize HLEC surface receptors and induce apoptosis via Rho kinase activation. When taken up by HLEC, the same soluble factors may also be able to directly activate Rho kinase. Our results also indicate that a given parasite strain induces HLEC apoptosis either by direct contact or via soluble factors, but not by both mechanisms. Indeed, PRBC that trigger apoptosis by cytoadherence did not trigger the release of soluble factors capable of inducing cell death. For example, isolate F528 was not apoptogenic after 24 h of contact coculture (Table 1), whereas it induced apoptosis via diffusible factors in non contact experiments (Table 2). Ho et al. 1991 have also demonstrated the inverse relationship between rosette formation and cytoadherence [17]. Hence *P. falciparum* field isolates may have a greater propensity for cytoadherence or rosette but not both. Transcriptome analysis of PRBC inducing HLEC apoptosis thorough cytoadherence has revealed that pathogenicity factors (PALPF) do not act exclusively as adhesins. Other transmembrane proteins and secretory-excretory molecules none involved in cytoadherence have also been identified [27], such as the trypanosome apoptogenic factor involved in human brain EC apoptosis [42], [43].

One of the most important findings of this study is that Fasudil (HA-1077) inhibits both mechanisms leading to HLEC death (cytoadherence and soluble factors), whereas it did not affect PRBC cytoadherence (Table 1). Fasudil inhibits Rho kinase phosphorylation of various substrates, including other kinases and enzymes involved in HLEC activation [32], [44], [45]. This inhibition of the Rho kinase signaling pathway may be sufficient to inhibit PRBC-mediated HLEC apoptosis mediated both by cytoadherence and by diffusible factors, assuming that Rhokinase is activated by both stimuli. Rho kinase inhibition by Fasudil also inhibits NO production. Low or physiological NO concentrations can exert an antiapoptogenic effect on several cell types, including EC [46], [47]. This antiapoptogenic effect of NO involves several types of stimuli including TNF alpha [48], [49]. Our results reinforced the hypothesis in that preventing EC activation could be exploited for CM treatment [50].

In conclusion, we show that the HLEC-*P. falciparum* interaction is more complicated than previously thought, and points to Rho kinase inhibition as a potential therapeutic intervention. These findings may warrant clinical trials of fasudil in severe malaria management.

## Materials and Methods

### Patients

Patients were enrolled in two local Gabonese hospitals (Centre Hospitalier Régional Amissa Bongo and Hôpital de l'Amitié Sino-Gabonaise, Franceville, Haut-Ogooué Province) between April and December 2008. The study was submitted and approved by the Ethic Committee of Bio-Medical Research of Franceville (Comité d'Ethique de Recherche Bio-Médicale de Franceville, CERB) and supported by the Gabonese Ministry of Health. Children with symptoms of acute malaria were recruited with their parents' or guardians' written informed consent. World Health Organization criteria were used to diagnose uncomplicated and severe malaria [51]. *P. falciparum* was detected by thick film (Lambaréné method) and thin film microscopy. The children were aged from 0 to 14 years and all had ≥5000 parasites/µl of blood. Blood was drawn into sterile EDTA tubes for culture. As compensation, all the children were treated free of charge with a combination artesunate and amodiaquin (Arsucam®), the first-line treatment recommended by the Gabonese authorities. Also, biomedical analysis (blood cells account, hematocrit determination etc.) was done freely for each child.

### Plasmodium falciparum culture

Thick and thin blood films were used for diagnosis. Thick films were prepared with Lambaréné's method: briefly, 10 µl of total blood was spread over a rectangle measuring 10 by 18 millimeters on a microscope slide [52]. Parasite load was calculated as the number of asexual forms of *P. falciparum* per µl of blood, as previously reported [53].

PRBC were also selected for their “viability”, because PRBC from patients having self-medicated (and who rarely inform their physician) do not grow in culture.

White cells were removed by 3 to 4 washes in RPMI 1640 incomplete culture medium. PRBC were then immediately cultured as previously described [54]. Briefly, RPMI 1640 medium was supplemented with 8.3 g/L HEPES, 2.1 g/L sodium bicarbonate, 0.05 g/L hypoxanthine, 0.1 mg/mL gentamicin, 1 µg/mL fungizone, 2 g/L D-glucose and 0.4% Albumax II (InVitrogen, Cergy Pontoise, France) and the hematocrit was adjusted to 5%. PRBC were then incubated at 37°C for 24–48 h. Mature PRBC were then enriched by gelatin flotation [55] and counted under a microscope.

### Culture of human lung endothelial cells (HLEC)

Ninth-passage HLEC derived from the same batch, as documented elsewhere [33], were maintained in flasks at 37°C with 5% CO2 in Medium 199 containing 10% (vol/vol) fetal calf serum, 50 units/ml penicillin-streptomycin and 0.25 mg of Fungizone (InVitrogen, Cergy Pontoise, France). Appropriate receptor expression (von Willebrand factor, ICAM-1, VCAM-1, CD31, CD36, E/P-selectin and CSA) was verified.

### Cytoadherence assay

HLEC were resuspended in Medium 199 and subcultured at a density of 3000 cells/well in 8-well tissue culture plates (Labtek) for 24 to 48 hours in standard conditions until confluence was reached.

Confluent HLEC were then used directly in adhesion assays, or kept at 4°C in 300 µL of phosphate buffered saline (PBS) for a maximum of 2 months after fixation for 20 minutes at 37°C with 2% paraformaldehyde [56]. A suspension of 300 µl of mature PRBC (approximatively 24×10^6^ schizont) was added to each well containing HLEC monolayers from Labtek in duplicate and incubated for 1 hour at 37°C. Unbound PRBC were removed by washing 3 times with PBS. The labtek was fixed for 20 minutes at room temperature with 2% glutaraldehyde, rinsed with PBS and stained with Giemsa. Adhesion was expressed as the number of PRBC adhering to 1000 HLEC (Table 1).

### Apoptosis assay

HLEC were subcultured in the lower compartment of 6-well Transwell plates (polyester 0.4 µM pores (COSTAR®, Corning Incorporated, NY USA) and in 96-well plates (Corning Incorporated, NY USA), until confluence. A suspension of 2.500 µl (around 200×10^6^) and 100 µl (around 8×10^6^) of each mature PRBC preparation in parasite culture medium was added to each well of Transwell plates and 96-well plates in duplicate and quadruplicate, respectively, and incubated for 4 h or 24 h at 37°C. HLEC were incubated with uninfected RBC as negative controls. Apoptosis was measured with an enzyme linked immunosorbent assay (ELISA) (Cell Death Detection ELISA^plus^, Roche Diagnostics, Mannheim, Germany).

After 4 hours of incubation in 96-well plates, PRBC and RBC were washed 3 times with RPMI 1640 and twice with PBS. The upper Transwell compartment containing PRBC and RBC was removed and the supernatant was discarded. Hundred µl and 2,500 µl of the lysis provided with the ELISA kit were used to permeabilize HLEC in each well respectively from 96-well plates and Transwells, followed by 30 min of incubation at room temperature. The plates were then centrifuged and 20 µl of supernatant per well was used in the ELISA. The assay measures intracytoplasmic nucleosomes (degraded DNA) released from apoptotic HLEC. Optical density (OD) was read using Stat Fax® 3200 (Fisher Bioblock Scientific, Illkirch France) at 405 nm with a reference filter of 492 nm. The mean OD ratio of PRBC-activated HLEC to RBC-activated HLEC was calculated for each PRBC preparation, using a positivity cut-off of 3, as recommended elsewhere [20], [26].

### Apoptosis inhibition assay

Before contact and non contact coculture, each PRBC suspension was treated with Fasudil at a final concentration of 30 µM.

### Statistical analysis

Data were analyzed with the chi 2 test and Student's *t* test. The Mann-Whitney *U* test was used for non normally distributed data. Significance was assumed at *P*<0.05.
